# Attitudes toward treating functional neurological disorders: psychological service provider perspectives

**DOI:** 10.3389/fpsyt.2026.1892903

**Published:** 2026-10-23

**Authors:** Robin Garrett, Leila Hoti, Nicole A. Roberts, Mary H. Burleson, Lorna Myers, Alan Wang

**Affiliations:** 1 University of Arizona College of Medicine, Phoenix and Banner University Medical Center, Phoenix, AZ, United States; 2 School of Social and Behavioral Sciences, Arizona State University, Phoenix, AZ, United States; 3 Northeast Regional Epilepsy Group, Hackensack, NJ, United States

**Keywords:** assessment, conversion disorders, functional movement disorder, functional neurological disorder, functional/dissociative/non-epileptic seizures, neuropsychology, psychotherapy

## Abstract

**Introduction:**

Functional neurological disorders (FND) are characterized by involuntary sensory, motor, and/or other seemingly neurological symptoms lacking expected medical explanation. Once diagnosed, patients often are referred to mental health providers for psychotherapy, which is effective in treating many FND symptom presentations either alone or as part of an integrative approach. Mental health providers may feel reluctant or unprepared to accept these referrals, however.

**Methods:**

We conducted a U.S.-based online survey of 286 practicing psychological service providers (psychologists, psychiatrists, counselors, social workers in private practice, neuropsychologists) to examine knowledge of, attitudes toward, and preparedness to treat FND.

**Results:**

Findings revealed that most of our sample had limited exposure to FND in routine practice. Confidence in treating FND was modest, with neuropsychologists reporting more knowledge of and more confidence in treating FND than other psychologists/mental health providers. Greater perceived adequacy of psychotherapy training for, and more experience working with, FND each were associated with more positive FND attitudes, including greater confidence and enjoyment in treating FND and greater belief that symptoms would improve.

**Discussion:**

This study is novel in its examination of knowledge and attitudes among psychological service providers, who are pivotal in furnishing effective treatment for FND patients but may not be well-versed in their needs. In contrast to existing studies of medical providers that revealed stigma as a major barrier to providing adequate care for FND, our findings suggest lack of knowledge and lack of access to FND patients may limit the ability to gain more training and expertise. Targeted training and consultation for mental health providers may improve confidence and motivation and expand the workforce capable of delivering effective treatment for FND.

## 1 Introduction

Functional neurological disorders (FND) are characterized by various symptoms including sensory, motor, and cognitive symptoms lacking expected medical explanation. In the *Diagnostic and Statistical Manual of Mental Disorders 5^th^ Edition (DSM-5-TR*), FND is listed as Functional Neurological Symptom Disorder, with Conversion Disorder listed parenthetically (1). There are two major or most common subtypes of FND (which are heterogeneous in presentation and can co-occur): Functional movement disorder (FMD) and functional/dissociative seizures (FDS). FMD consists of movement symptoms without distinctly identifiable neurological origin, and diagnosis requires a physical examination from a neurologist trained in movement disorders (2). In addition to information from a neurologist’s examination, diagnosis of FDS (also known as psychogenic non-epileptic seizures) requires the presence of seizure-like behaviors without epileptic cortical activity during the FDS events based on video electroencephalogram (vEEG) (2, 3). Per a recent review, estimated FND incidence is 10-22/100,000 and estimated minimum prevalence is 80-140/100,000, with an estimated prevalence range of 50-1,600/100,000 (4). Because FND symptoms outwardly mimic neurological ones, patients typically present for diagnosis and treatment in neurology clinics or are referred to neurologists from emergency departments (5). Although prognosis for FND is generally unfavorable, younger age of onset and earlier diagnosis after symptom onset are associated with better outcomes (6), and treatment can be promising as discussed below.

After an FND diagnosis is confirmed, the neurologist must communicate sensitively that the condition is FND (7) rather than the presumed counterpart neurological condition, and that psychological treatment is recommended – either on its own or as part of an interdisciplinary approach. The patient is then referred to mental health professionals for psychiatric care and psychotherapy. This is often counterintuitive, as the observed FND symptoms (e.g., seizures, tremors, paralysis) are not inherently associated with psychotherapy. Thus, even after addressing the major challenges of 1) diagnosing FND and 2) facilitating the patient’s acceptance of the diagnosis and willingness to seek psychological services, an equally serious challenge arises: determining and ensuring the availability of knowledgeable mental health providers willing and able to treat FND. As discussed below, there are a host of reasons why psychologists and other mental health providers may be reluctant to treat FND, including both a lack of knowledge and preconceived notions about these conditions.

Systematic examination of mental health provider perspectives is essential, because without understanding how to create an adequate mental health treatment pipeline, patients may return to their neurologist and/or otherwise remain in a cycle without proper treatment. To address this issue, we conducted a survey to investigate U.S.-based mental health providers’ knowledge of FND, services provided for FND, attitudes toward FND patients, and FND-related training experiences and attitudes.

### 1.1 Psychotherapy for FND

The key impetus for assessing psychological service providers’ knowledge of and attitudes toward FND is that psychotherapy, either as a stand-alone treatment (especially for FDS) or in the context of interdisciplinary treatment modalities (e.g., including physical, occupational, and/or speech therapy for FMD), is essential for FND treatment [reviewed in (8) and (9)]. Although treating comorbid conditions with psychiatric medication may improve quality of life and stress, there is no current medication to directly ameliorate FND symptoms (10, 11).

For FDS, which is the most common subtype of FND, randomized controlled trials (RCTs) have pointed to significant improvement with psychotherapy, specifically cognitive behavioral therapy (CBT) and neurobehavioral therapy (NBT) (12, 13) see (14) and (15) for review and meta-analysis). For example, FDS-tailored CBT has improved FDS frequency and severity, quality of life, and comorbid psychological conditions (13, 16, 17). The large multi-site Cognitive Behavioural Therapy vs Standardised Medical Care for Adults with Dissociative Non‐Epileptic Seizures (CODES) trial found greater treatment effectiveness after a 12-month follow up when CBT was added to Standard Medical Treatment versus Standard Medical Treatment alone, with respect to period of time without seizures (albeit without differences in seizure frequency) and subjective quality of life indicators (e.g., less bothered by seizures, less impact of seizures on functioning) (18–21). Other psychotherapy approaches also have shown evidence of effectiveness, including Retraining and Control Therapy (ReACT) for pediatric FDS (22), mindfulness-based therapy (23), psychoeducation (24), psychodynamic therapy (25), interdisciplinary brief psychotherapeutic intervention (26), and trauma-focused therapy, including based on pre-RCTs and a small RCT of prolonged exposure (27–29). Whereas for FDS, physical therapy and physiotherapy interventions typically would not be recommended unless there are additional motor or sensory symptoms, these latter treatments are part of a first-line approach for FMD (2, 30). For FMD, randomized feasibility and clinical trials demonstrate treatment efficacy with physiotherapy and a combination of CBT plus physiotherapy interventions [ (2, 31, 32); also reviewed in (33)]. For example, in the Physio4FMD RCT, although there were not differences in the primary outcome of physical functioning, physiotherapy was associated with greater perceived or subjective control over motor symptoms (59% of patients working with a physiotherapy specialist reported improvement after 12 months versus 38% given treatment as usual), alongside improvement in subjective indicators of quality of life and functioning (34, 35).

### 1.2 Perceptions of FND

#### 1.2.1 A need to focus on mental health provider perspectives

Much of the research examining healthcare provider perspectives and experiences with FND has focused on physicians, primarily neurologists/epileptologists and psychiatrists [reviewed in (36) and (37)]. While these studies provide invaluable insight as to the clinical challenges that arise when treating patients with FND, they often overlook the roles of most mental health care providers. Mental health provider perspectives are critical to include, because once neurological conditions are ruled out and FND is diagnosed, mental health providers are responsible for implementing intricate treatment plans involving psychotherapeutic intervention, which is the treatment of choice for FDS and integral to an interdisciplinary approach for FND. A small qualitative study underscored psychotherapists’ challenges working with FDS patients after they have undergone an often-lengthy and confusing diagnostic process; furthermore, therapists lack clear guidance from the field regarding how to meet the individual treatment needs of this heterogeneous patient group (38). This is in contrast to the substantial efforts by the neurology community to train their providers in FND, and to provide guidance in working with this population. These prior findings provide an important foundation for the current study, which addresses perceptions with a larger range of providers.

#### 1.2.2 Negative healthcare provider attitudes toward FND

Negative healthcare provider attitudes towards patients with FND (e.g., that patients are difficult or unpleasant to work with, both in general and compared with other patients) have been documented across specialties within medicine and, albeit to a lesser extent, psychology (39–41). Although the literature surrounding FND has changed over time, with neurologists and other specialties more knowledgeable about FND as a legitimate neuropsychiatric condition, many health care professionals still view FND as less legitimate than neurological conditions and often attribute symptoms to malingering, manipulation, attention seeking, or disability seeking (41–44). These attitudes are demonstrated based on implicit as well as explicit measures among a heterogeneous sample of healthcare providers (e.g., combining neurologists, psychiatrists, psychologists, and others) and are associated with lower likelihood of making appropriate referrals (39). Thus, this stigma may have real consequences for who is willing to treat, or even gain knowledge about how to treat, FND.

### 1.3 Role of mental health professionals

Mental health professionals (e.g., psychologists, psychiatrists, mental health counselors, clinical social workers) who are trained to provide psychotherapy to treat emotional, behavioral, psychological, interpersonal, or other difficulties can be in a strong position to treat FND. Different modalities, therapeutic techniques, and specific tools that providers already may deploy in their day-to-day practice likely apply to FND. For example, in addition to developing a narrative of the patient’s history, treatments for FDS typically include psychoeducation, symptom monitoring with seizure logs, sensory grounding (e.g., to prevent the onset of an episode), emotion and/or trauma processing, cognitive restructuring, mindfulness, and other strategies for emotion regulation, self-care, and problem-solving (45). Knowledge that psychotherapy can help alleviate FND symptoms across theoretical orientation and provider type (8) could pave the way for mental health providers who may not ordinarily treat FND to provide these services.

### 1.4 Role of neuropsychologists

Neuropsychologists, who are trained as clinical psychologists while also receiving specialized training in brain-behavior relationships and the assessment of those relationships, play a unique role for FND patients. They often work closely with neurologists, including working in medical settings with epileptologists and movement disorder specialists, and therefore may be more likely than other types of psychologists and mental health providers to encounter individuals with FND and serve as a point of contact for psychoeducation and referrals. Additionally, patients of any kind reporting cognitive complaints would likely be referred to a neuropsychologist for testing. Given that the primary role of neuropsychologists is to provide assessment rather than ongoing psychological treatment including psychotherapy, FND patients referred to a neuropsychologist would likely need to be referred again to a mental health provider for therapy, perpetuating the cycle of multiple referrals and providers. To the extent that neuropsychologists have received training that allows them to provide therapy or otherwise feel equipped to address FND symptoms, and to the extent that their role (e.g., job responsibility allocation) builds in time for the provision of therapy, they could provide an additional and important avenue for patient care.

### 1.5 Training for treatment of FND

A large majority of patients are referred by neurologists to mental health providers in the community with the expectation that these providers understand FND and are equipped to effectively manage the care of these patients. These providers may not believe they have adequate training in FND to competently treat the complexity of this population. This may lead to patients re-presenting to neurology due to continued FND symptoms despite efforts to pursue mental health treatment. Thus, this can lead to helplessness and frustration for the neurologist and for the patient.

While mental health providers are typically trained in CBT, psychodynamic therapy, mindfulness, or other psychotherapeutic approaches that may be applicable to FND, their exposure to FND is often limited. Although psychodynamically-oriented training programs in the past may have discussed the concept of conversion symptoms and disorders, and training programs that emphasize a medical model may discuss diagnostic (*DSM*) criteria for somatic symptom disorders, a gap remains in bridging to current thinking about FND. To our knowledge, most clinical and counseling graduate programs and even clinical internships in the United States do not provide training experiences focused on FND, which can result in less enthusiasm for working with this population and/or psychologists viewing themselves as unprepared to work with this population.

Psychologists familiar with traditional diagnostic nomenclature and concepts could be expected to have knowledge of the Freudian notion of Conversion Disorder and of other mental health-related illness presentations [e.g., somatic symptoms associated with anxiety and depression; bodily symptoms in populations that express distress somatically (46)]. They may not know to consider applying this knowledge when encountering or working with FND patients, especially if their training settings did not offer exposure to these patients. Even in a mental health context, neurological-seeming behaviors (e.g., seizures, tremors) are likely to be taken at face value and perceived as requiring neurological/medical intervention.

### 1.6 Current study

The current study is one of few that investigates mental health providers’ knowledge of, extent of training in, and comfort in providing treatment to patients with FND. We assessed this online in a national (primarily U.S.-based) sample through providers’ quantitative ratings and open-ended responses.

We anticipated that participants would report, first, limited knowledge of and experiences with FND and, second, mean ratings reflecting negative attitudes toward working with FND patients, including negative expectations, perceptions that FND patients are challenging to treat, a belief that FND symptoms are unlikely to improve, and minimal confidence in treating FND. Third, we expected that participants would report feeling minimally prepared based on their training to provide psychotherapy for FND and would report barriers to training, such as a lack of access to available training opportunities. Finally, we expected that more FND knowledge, experience, and perceived training preparedness would be associated with less negative and more positive attitudes toward working with FND.

Additionally, given the role of neuropsychologists in bridging neurology and psychology, we compared neuropsychologists with other (i.e., non-neuropsychologist) psychologists/mental health providers, and explored alternative possibilities that neuropsychologists would either report more positive attitudes toward FND than other mental health providers, given greater training and knowledge, or would report more negative attitudes, given working more closely with medical providers where there may be more stigma as documented previously (36).

**Figure 1** illustrates a framework for how the complex factors involved in FND as described above may come together to hinder the provision of psychological treatment for FND. For example, patients may not be referred to psychologists/other mental health providers, and if they are, providers may not feel they have the needed expertise, may believe that FND reflects an underlying medical condition requiring a different set of providers, and/or may have other perceptions or concerns that result in the turning down of referrals that do come to their practice.

**Figure 1:**
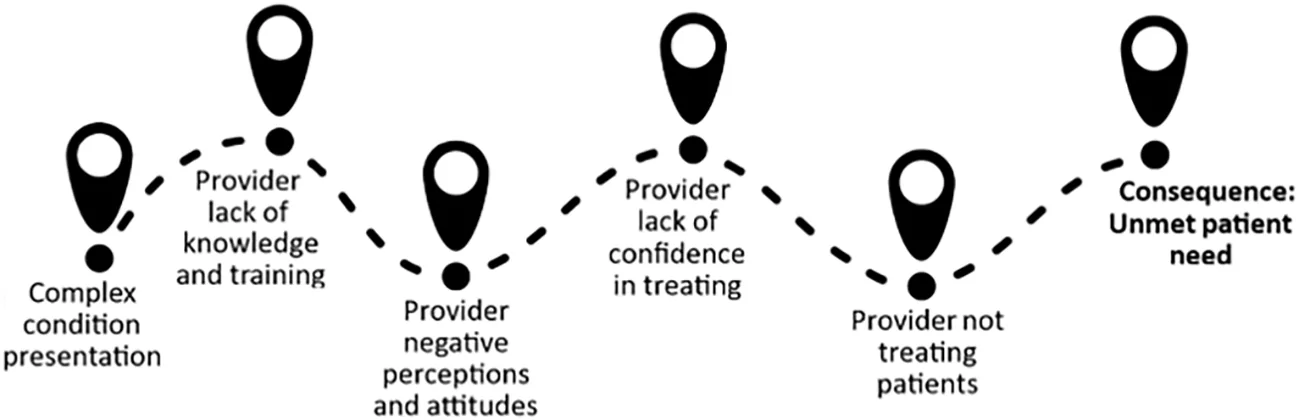
Possible barriers to psychological treatment for FND. Roadmap for how the complex factors involved in approaching FND may come together to hinder the provision of psychological treatment for FND. These factors, including provider training and attitudes, were assessed in the current survey and suggest areas of intervention.

## 2 Methods

The study aims and methodology were approved by the Institutional Review Board; below we follow STROBE sample reporting guidelines for cross-sectional observational studies (47). Our focus was on providers with psychologically-based clinical training experiences who were in practice and providing mental health treatment or assessment.

### 2.1 Recruitment and participants

Our recruitment strategy comprised emails, listserv announcements, and direct outreach aimed at mental health providers nationwide in a variety of settings (e.g., behavioral health hospitals, academic hospitals, outpatient clinics, private/group practice) and with different approaches and roles (e.g., trauma therapists, neuropsychologists, CBT practitioners). Recipients were asked to pass the information to other relevant individuals.

To encourage participation across the possible range of FND knowledge, recruitment materials included this statement, “Even if you have no knowledge about FNDs, you are invited to participate, as we are interested in learning about the scope of knowledge and understanding across a range of providers.” These methods captured psychologists and other kinds of mental health providers (e.g., counselors, clinical social workers) but also resulted in survey completion from those in adjacent fields outside of psychology/mental health.

Of the 473 respondents who initially began the survey, 134 did not meet inclusion criteria, namely having a clearly indicated psychology and/or psychotherapy background or practice, and were excluded prior to any analysis. They comprised 98 physical therapists, two occupational therapists, two psychometrists, three physicians (one epileptologist, two double- or multi-board certified in neurology and other specialties), two nurses, three unspecified “other,” seven trainees, and 17 who did not identify their provider type. Of the remaining 339 respondents, we excluded 53 who did not provide data through and beyond the FND definition. Our final sample of 286 participants included 177 neuropsychologists, 77 psychologists, 20 counselors, seven social workers in private practice, and five psychiatrists. For comparisons of neuropsychologists with other mental health practitioners providing care for FND, we combined the latter four groups (*n =* 109). We note that all of the participating counselors, social workers, and psychiatrists either worked in private/group practice or community mental health settings and/or were providing psychotherapy to FND patients in those or other settings. Although all psychologists/neuropsychologists were not necessarily providing psychotherapy to FND patients at the time of the survey, they offered therapy, assessment, or other related services and presumably received the theoretical and practical training relevant to testing our study aims.

### 2.2 Measures

The online survey comprised questions regarding participants’ demographics, professional background, and practice characteristics; their attitudes toward and knowledge of FND and its treatment; their desired and completed education and training for FND; and perceived barriers to that training. Items were adapted from previous similar studies that focused primarily on medical providers (e.g., neurologists/epileptologists) (40, 43, 48–55). Our goal was to maximize participation of full-time psychological service providers; we therefore followed prior similar studies [e.g (40)] and minimized time burden by using single, face-valid items to assess FND knowledge, attitudes, and training.

#### 2.2.1 Provider and practice characteristics

Participants reported their profession (i.e., provider type), years in practice, practice setting, and geographic region, as well as age, gender, and racial/ethnic background.

#### 2.2.2 FND definition knowledge

An initial open-ended question assessed knowledge of FND as follows: “To your knowledge, what is a ‘functional neurological disorder’ (FND)? Also, to your knowledge, what are some examples? If you do not know, please say don’t know.”

First, we coded as “0” responses where participants made explicit mention of uncertainty or not knowing what FND is (e.g., “I don’t know” or “I’m not sure”), without providing any additional information. Second, for any attempt at a definition, we rated level of understanding as “1” = minimal/weak understanding, “2” = moderate or “so-so” understanding, or “3” = strong understanding. A trained undergraduate research assistant and a trained graduate student (co-author LH) each coded the definitions. Both individuals coded all responses. Percent agreement was 77%, with a weighted kappa of.760. For definitions on which there were disagreements, they were resolved via discussion and adjudication by a third reviewer (co-author NR). Analyses with these ratings used a final scale of 0 to 3, where 0 reflected responses of “I don’t know” without a definition or further information provided, and 1, 2, or 3 were the aforementioned codes (weak, moderate, strong) for any definition (or speculation) provided. Finally, concepts and terms that frequently emerged in participants’ definitions were coded (e.g., mention of conversion disorder, mention of FND as a disorder of exclusion), with a “1” assigned if present. Examples of definitions assigned different codes are included in the **Supplementary Materials**.

After participants provided their own definition, the survey presented the following brief FND definition on which participants could anchor their remaining survey responses: “Functional neurological disorders (FND), such as psychogenic nonepileptic seizures (PNES), historically have been described as conversion disorders. They are symptoms that may appear neurological but do not have the expected medical/neurological basis. In PNES, for example, the person shows seizure-like symptoms yet does not have epilepsy or any brain-based signs of a seizure.”

#### 2.2.3 Experience with FND patients and services provided

Participants used a slider with percentages from 0 to 100 to indicate percentage of their patients with FND. Using checkboxes (multiple selections were allowed), participants indicated if they provided psychotherapy, psychological assessment, neuropsychological assessment, medication management, and/or other treatment to FND patients, as well as if they provided FND patients with referrals to other providers for psychotherapy or medication management. They also used checkboxes (again, with multiple selections allowed) to indicate the type of psychotherapy they provided for FND (e.g., cognitive-behavioral therapy, trauma-focused therapy), including an option for “other therapy.”

#### 2.2.4 Attitudes toward FND

Attitudes toward FND were assessed with a series of items developed for this study based on themes and items adapted from previous studies as noted above. Participants were asked five questions on a Likert scale (1=strongly disagree to 5=strongly agree) regarding whether they found patients with FND to be 1) “more challenging to work with than other patients,” 2) “more enjoyable to work with than other patients; and whether they 3) “get concerned that FND patients’ symptoms are in fact neurological/medical,” 4) “feel that patients with FND should be working with a medical provider regarding their symptoms instead of a psychological services provider”; and 5) “have negative expectations about working with FND patients.” They also rated their confidence in treating FND patients on a 1 (not at all confident) to 5 (very confident) scale and rated, “To what extent do you believe patients with FND can have symptom improvement?” on a scale from 1 (not at all) to 5 (very much).

After asking participants to rate these items regarding attitudes toward FND, we provided an opportunity in an open-ended fashion: “Please indicate anything else you would like to mention regarding working with patients with FND.”

#### 2.2.5 Training for FND

FND training perceptions, experiences, interest, and barriers were assessed. First, participants were asked to rate (1=little to no adequate training to 5=very well trained) whether they believed they had adequate training to provide psychotherapy, medication management, psychological assessment, or neuropsychological assessment, respectively, for patients with FND. Second, using checkboxes, participants were asked to indicate the types of FND training previously received (e.g., via graduate coursework, during a practicum rotation; multiple selections were allowed). Third, participants rated (1=not at all interested to 5=very interested) their interest in specific kinds of training for FND (e.g., professional conference presentations, private supervision/consultation). Fourth, participants rated (1=not at all interested to 5=very interested) in retrospect how interested they would have been in each of several FND training opportunities at the time of their educational training (e.g., graduate coursework, rotation on an epilepsy monitoring unit/in a movement disorders clinic). Finally, participants rated how much each of several items would be a barrier to obtaining education in FND at present (1=not a barrier at all to 5=a very large barrier) and were prompted to respond in an open-ended fashion to the item, “Please indicate any other barriers you envision to your obtaining education in functional neurological disorders now.”

### 2.3 Procedure

After providing informed consent, participants completed a brief (designed to be completed in ~10 minutes) online survey administered via Qualtrics.com, a secure data collection platform. The survey was completed anonymously. Participants were given the option of providing their contact information at the end of the survey (linked separately) for entry into a drawing for one of two $50 gift cards. All procedures were approved by the university’s Institutional Review Board and participation was voluntary.

### 2.4 Data analysis

Data were analyzed using SPSS Statistics software (v29.0.2). Given the study aims, results are largely descriptive (i.e., summaries of quantitative and categorized open-ended responses), with bivariate Pearson product-moment (*r*) correlations used to test associations between attitudes toward FND patients and providers’ FND knowledge, training, and experience (with pairwise deletion of missing data). Spearman’s *rho* (*ρ*) correlations are also reported for reference, given the use of single-item measures. To compare background, experiences, and attitudes between psychologists/mental health providers and neuropsychologists, univariate analysis of variance (with partial eta squared as a measure of effect size) was used for continuous variables, and chi-square and *post hoc* pairwise tests of proportions (with *z*-values representing adjusted standardized residuals for the latter) were used for categorical variables. For these comparisons, family-wise corrections for multiple tests used the Holm’s sequential Bonferroni procedure, which has been used in neuropsychological research and is less conservative than the traditional Bonferroni procedure (56).

## 3 Results

### 3.1 Provider and practice characteristics

Characteristics including provider demographics, practice type, practice setting, and percentage of FND patients treated are in **Table 1** and **Supplementary Table S1**. While the majority of providers in our sample were women (70.7% of those reporting gender) and non-Hispanic White (77.9% of those reporting ethnic/racial background), providers reflected a range of backgrounds, practice settings, and U.S. geographic regions. Recruitment efforts focused on the U.S., given potential country-level differences in training and healthcare implementation, but we included 11 participants (3.8%) who completed our survey and were practicing internationally. Participants worked most commonly in private practice (39.9%) or academic hospitals (28.3%), but again a range of practice settings were reported (see **Supplementary Table S1**).

**Table 1:** Provider demographics, profession, and practice characteristics.

Characteristic	*n*	*M*	*SD*
Age in years (range 25 to 75)	258	43.6	11.4
Years in practice (range <1 to 44)	286	11.9	10.6
Percentage (%) of FND patients	275	13.3	16.7

Characteristic	*n*	Subgroup %^a^	Total %^b^
Gender / sex	276		
Female	195	70.7	68.2
Male	79	28.6	27.6
Non-binary	2	0.7	0.7
Ethnicity / race	276		
Non-Hispanic White	215	77.9	75.2
Asian / Asian American / Pacific Islander	20	7.2	7.0
Hispanic / Latino	13	4.7	4.5
Black / African American	6	2.2	2.1
Biracial / Multiracial	11	4.0	3.8
Provider type / profession	286		
Neuropsychologist	177	61.9	61.9
Psychologist^c^	77	26.9	26.9
Counselor^c^	20	7.0	7.0
Social worker^c^	7	2.4	2.4
Psychiatrist^c^	5	1.7	1.7
Practice setting: Geographic region	286		
Southwest	75	26.2	26.2
Midwest	71	24.8	24.8
West	49	17.1	17.1
Northeast	42	14.7	14.7
Southeast	38	13.3	13.3
Non-U.S.	11	3.8	3.8

Percentages within characteristic may not add to 100 due to missing data/participants preferring not to answer.

^a^ Subgroup % = percentage of participants who reported data for that characteristic.

^b^ Total % = percentage of full sample, whether or not they reported the category (*N* = 286).

^c^ Grouped as psychologist/mental health provider (*n* = 109; 38.1%) for analyses comparing with neuropsychologists (see **Tables 7**–**9** and **Table S5**).

#### 3.1.1 Knowledge of FND: definitions

To assess participants’ knowledge of FND, as noted above, prior to providing them with a correct definition of FND, we asked participants to define FND “to their knowledge.” Seven percent (*n* = 20) reported not knowing what FND was and did not offer any further information (e.g., “I don’t know” or “?”). Of the 93% of the sample who provided a definition, close to half (45%) indicated a relatively strong understanding of FND, and the other half (55%) conveyed a moderate or relatively weak understanding. (See **Table 2**).

**Table 2:** Providers’ FND understanding based on their FND definition.

Coding of participants’ FND definitions	*n*	%
For all responses to request for definition of FND (*N* = 285)		
Did not provide a definition: reported not knowing or too uncertain to guess (code = 0)	20	7.0
Indicated minimal/weak understanding (code = 1)	60	21.0
Indicated moderate/“so-so” understanding (code = 2)	85	29.7
Indicated strong understanding (code = 3)	120	42.0

Coding of participants' FND definitions	*M*	*SD*
Mean level of FND understanding (coded 0-3)	2.1	1.0
Themes or terms mentioned in definitions	*n*	*%*
Conversion disorder	52	18.2
“Pseudo” ^a^	20	7.0
Secondary gain / malingering	6	2.1
Functional cognitive disorder / cognitive symptoms	54	18.9
FND as a disorder of inclusion (“positive signs”)	5	1.7
FND as a disorder of exclusion	5	1.7
Other non-FND-specific psychological or medical terms / disorders	63	22.0

Statistics are based on full sample (*N* = 285; one participant had missing data for this item). Participants could use more than one theme or term in their open-ended definition of FND.

^a^ 14 “pseudoseizure,” 6 other “pseudo-” conditions (e.g., “pseudotremor”).

#### 3.1.2 FND caseload, treatment, and referrals

Providers indicated the full range of possible percentages of FND patients on their caseload (0% to 100%). Twenty-three participants (8%) reported that none of their patients had FND; one participant reported that 100% of their patients had FND (a participant who was providing an FND-specific treatment). Most of the sample saw relatively few FND patients on a regular basis, but the majority (*n* = 227 or 79.4%) reported providing psychotherapy, psychological assessment, and/or neuropsychological assessment for FND, indicating that to the extent they did see any clients with FND, they were providing direct psychologically- and/or neuropsychologically-relevant services.

**Supplementary Table S2** presents the number and percentage of unique participants who provided psychological assessment, neuropsychological assessment, and/or psychotherapy to patients with FND. Services and referrals provided for FND, reflected as counts of overlapping categories (e.g., assessment, therapy, other treatment modality) are listed in **Table 3**. Specific psychotherapy modalities, based on survey categories provided and write-in responses, are listed in **Supplementary Table S3**. These responses revealed a range of additional approaches provided for FND, including interpersonal, family, and group therapy, work with multidisciplinary teams, and psychoeducation for FND patients and their families.

**Table 3:** Psychological services and referrals provided for functional neurological disorder (FND).

Treatments, services, and referrals	*n*	Subgrp %	Total %
Provide any FND treatment/psychological services			
Psychotherapy^a^	108	47.6	37.8
Psychological assessment	71	31.3	24.8
Neuropsychological assessment	157	69.1	54.9
Medication management	3	–	1.0
Other treatment modality (open-ended)^b^	–	–	–
Psychoeducation / family support during assessment feedback	4	–	1.4
Interdisciplinary co-sessions with occupational, physical, and/or speech and language therapy	2	–	0.7
Cognitive rehabilitation therapy / compensatory strategy training	1	–	0.4
Offer referrals for FND			
Psychotherapy	102	–	35.7
Medication management	84	–	29.4
Neuropsychological assessment^c^	2	–	0.7
Physical therapy, occupational therapy, speech and language pathology, and/or FND-specific functional disability-focused referral^c^	2	–	0.7
Neurology^c^	1	–	0.4
Do not see enough FND patients to answer	40	–	14.0

Subgrp % = percentage of subgroup, where subgroup comprises participants who provided data for the category or category subgroup of items reported or analyzed per table section. Total % = percentage of full sample, whether or not they reported the category (*N* = 286). Across all items, percentages exceed 100 because participants could select multiple options.

^a^ Yes-no item regarding whether participant provided psychotherapy to patients with FND, either alone or in combination with other treatment.

^b^ Open-ended question regarding provision of other treatment not specifically listed (no checkbox was presented).

^c^ Not presented in the survey as referral options, but participants specified in response to the “Other Treatment” prompt.

As shown in **Table 3**, participants also reported referring FND patients to other providers for psychotherapy and/or medication management, and participants offered additional kinds of providers to whom they referred, including neurology, physical or occupational therapy, and/or speech and language pathology. One CBT therapist reported referring for trauma therapy.

#### 3.1.3 FND training experiences and barriers

Provider FND-related training experiences are listed in **Supplementary Table S4**. Most training experiences were self-initiated, informal, or time-limited, with relatively fewer participants reporting FND-related coursework or a formal rotation on an epilepsy or movement disorders unit.

Ratings (1 to 5 scale) assessing participants’ attitudes and beliefs about training for FND are shown in **Table 4**. Providers in our sample reported feeling more prepared to provide general psychological assessment or neuropsychological assessment than therapy for FND, *t (278*) = 9.78, *p* <.001, and *t (278*) = 7.64, *p* <.001. Participants also rated retrospectively their level of interest in different kinds of FND training, should such opportunities have been available. With respect to current training interests, mean ratings suggested that participants favored any training opportunities that offered continuing education credits, followed by professional conference presentations on mechanisms of or psychotherapy treatment for FND. See **Table 4** for means and standard deviations.

**Table 4:** Functional neurological disorder (FND) training attitudes and beliefs.

Training attitudes and beliefs	*M^a^*	SD
Perceived FND training preparedness (*n* = 279)		
Psychological assessment	3.5	1.4
Neuropsychological assessment	3.5	1.6
Psychotherapy	2.7	1.3
Medication management	1.2	0.7
FND training: Retrospective interest (*n* = 276)^b^		
Internship and/or fellowship rotation on psychological treatment for FND	3.6	1.4
Internship and/or fellowship with epilepsy or movement disorders rotation	3.6	1.4
Graduate program coursework in FND	3.5	1.3
FND-focused practicum	3.1	1.3
FND training: Current interest (*n* = 277)		
Any training offering continuing education credits	4.1	1.0
Professional conference presentations: Pathophysiology/ mechanisms	4.0	1.1
Conference presentations: Psychotherapy treatment	3.9	1.2
Community-based trainings	3.3	1.3
Private supervision/consultation	3.1	1.3
Perceived barriers to current FND training (*n* = 276)		
Time: Taking time from practice	3.3	1.3
Time: Not enough time outside work	3.2	1.3
Cost	3.1	1.2
Access limitations: Location	3.1	1.2
Lack of interest	1.9	1.1
Access limitations: Journals/resources	1.6	1.0
Access limitations: Online training	1.2	0.6

Training attitudes and beliefs	*n*	%^c^
Other barriers (open-ended)		
Low number of patients in provider’s practice (including results in low motivation to learn, does not justify time or cost, yields minimal opportunity to practice skills gained)	9	3.1
Lack of awareness/knowledge of available trainings (including buried in high volume of other professional announcements)	7	2.4
Limited supervisors available	5	1.7
Conflicting evidence / lack of empirically supported evidence	2	0.7
Potential non-belief in the diagnosis	1	0.3

^a^ Items were rated on a 1 to 5 scale with higher values reflecting greater endorsement of the item.

^b^ Level of interest participants would have had in taking this training at time of their primary education and training.

^c^ Percentages are calculated from the full sample (*N* = 286).

Based on mean ratings, cost, time, and access to in-person training locations were the top three barriers to obtaining training about FND at present; however, these mean ratings were not particularly elevated (see **Table 4**). Rather, the low volume of FND patients encountered was stated repeatedly in participants’ open-ended responses as a barrier to obtaining further training. Several participants also noted a lack of available supervisors for FND cases. Thus, the cost-benefit ratio may be a barrier to training for providers with a low number of FND patients, rather than a lack of FND interest or training opportunities per se.

### 3.2 Attitudes toward FND

Means, standard deviations, and intercorrelations among provider attitudes toward working with FND patients (seven single items) are presented in **Table 5**. Attitudes were related in the expected directions, with the two more disparate items (i.e., concern symptoms might be neurological/medical, and belief that a medical provider would be better than a psychological services provider) less consistently related to positive and negative (affective/emotion-related) attitudes. Providers on average endorsed the belief that symptoms can improve, which was the most strongly-endorsed attitude. Although they perceived working with FND patients as challenging, they did not report high negative expectations with respect to working with FND patients.

**Table 5:** Means, SDs, Pearson, and Spearman intercorrelations among provider FND attitude items.

FND attitude item		1	2	3	4	5	6	7
1	Believe symptoms can improve		-.13* -.08	-.05 -.06	.38*** .38***	.17** .12	-.23*** -.22***	-.16* -.19**
2	More challenging to work with than other patients			.13* .10	-.20** -.18**	-.39*** -.36***	.44*** .41***	-.01 -.04
3	Concerned symptoms may be medical or neurological				-.27*** -.28***	.06 .07	.10 .08	.25*** .23***
4	Have confidence in treating					.34*** .29***	-.39*** -.40***	-.21*** -.27***
5	More enjoyable to work with than other patients						-.41*** -.44***	-.03 -.04
6	Have negative expectations of working with							.13* .14*
7	Believe patient should work with medical vs. psychological provider							
Mean		4.0	3.8	3.1	2.9	2.8	2.7	2.1
*SD*		0.8	1.0	1.0	1.1	0.9	1.1	0.8

Spearman statistics listed below Pearson statistics. *n* = 254 to 279. Individual items rated on 1–5 scales where higher values reflect greater endorsement of the item.

*p <.05. **p <.01. ***p <.001.

#### 3.2.1 Associations between FND experiences and FND attitudes

Findings were generally consistent with our expectation that more FND knowledge and experience with FND patients would be associated with fewer negative attitudes and more positive attitudes held by providers (see **Table 6**). We note that the patterns of significance and correlation coefficient magnitudes remained highly similar when excluding the 23 participants reporting that 0% of patients in their practice had FND. We also note that attitudes were not related to the provider’s number of years in practice (all *p*s >.05), with the exception that providers with more years in practice reported less of a belief that a medical provider was better suited to address FND than a psychological services provider, *r (262*) = -.15, *p* = .016; *ρ (262*) = -.18, *p* = .004.

**Table 6:** Pearson and Spearman correlations between provider FND experiences/knowledge and attitudes toward FND.

Attitude / knowledge items	Percentage FND patients in practice	FND know-ledge based on definition^a^	Perceived psycho-therapy training adequacy	Perceived psych assessment training adequacy	Perceived neuropsych assessment training adequacy
Believe symptoms can improve	.18** .17**	.26*** .25***	.28*** .28***	.19** .18**	.22*** .21***
More challenging to work with than other patients	-.09 -.02	.04 -.02	-.14* -.14*	-.03 -.02	.07 .04
Concerned symptoms may be medical or neurological	-.06 -.12	-.15* -.13*	-.17** -.16*	-.21*** -.22***	-.18** -.20**
Have confidence in treating	.43*** .45***	.27*** .27***	.56*** .56***	.47*** .46***	.35*** .37***
More enjoyable to work with than other patients	.22*** .07	-.01 -.01	.26*** .25***	.05 .04	-.10 -.08
Have negative expectations of working with	-.15* -.12	.02 .02	-.40*** -.41***	-.14* -.17**	.04 .01
Believe patient should work with medical vs. psychological provider	.06 -.01	-.12 -.09	-.08 -.13*	.03 .01	.03 .02
Percent FND patients in practice	–	.12* .20***	.33*** .25***	.25*** .27***	.21*** .31***
FND knowledge based on definition^a^	–	–	.13* .12*	.38*** .32***	.50*** .44***

*n* = 252 to 279. Spearman statistics listed below Pearson statistics.

^a^ FND knowledge based on coding of participant’s open-ended definition (0 = “don’t know” per participant; 1 = weak understanding, 2 = moderate understanding, 3 = strong understanding).

*p < .05. **p < .01. ***p < .001.

#### 3.2.2 Associations between perceived training adequacy and FND attitudes

Participants who perceived that their prior training prepared them to provide psychotherapy to patients with FND reported that they were more confident and had fewer negative expectations in working with FND patients, found them more enjoyable and less challenging to work with, and held a greater sense that symptoms can improve. They also rated less concern that symptoms were actually neurological/medical (see **Table 6**). Findings were similar but showed fewer significant associations with respect to perceived adequacy of training to provide psychological or neuropsychological assessment for FND.

#### 3.2.3 Provider attitudes and perspectives in open-ended responses

After asking providers to rate items assessing their attitudes toward FND, we asked them to indicate anything else they would like to mention regarding working with patients with FND. Approximately half of the sample (*n* = 153 or 53.5%) responded to this item. Many of the responses echoed participants’ ratings, in terms of expressing a belief that symptoms can improve and that those with FND are both enjoyable and challenging to treat [also consistent with prior qualitative findings described in (44)]. Comments underscored many of the systemic challenges facing those with FND and the consequences for mental health service providers, particularly those delivering psychotherapy and/or assessment. Many comments reflected a lack of awareness regarding established treatment protocols for FND, and, even with awareness of such protocols, there was a reported lack of guidance regarding how to access them easily and how to tailor them to a heterogeneous range of FND clients. Other providers in our sample expressed a lack of contact with FND patients, and a tone of ambivalence or neutrality was more salient across responses than one of criticality or negativity. Multiple comments reflected possibilities for addressing gaps that otherwise perpetuate unmet patient need, such as via neuropsychologists as noted below (see Discussion). These and other themes and examples are provided in the **Supplementary Materials**.

### 3.3 Provider type comparisons: neuropsychologists and psychologists/mental health providers

While many neuropsychologists provide psychotherapy or other forms of intervention in addition to offering testing and recommendations, we expected that FND knowledge and training experiences would differ between neuropsychologists and non-neuropsychologists (described here as psychologists/mental health providers). Age, years in practice, and gender distribution did not differ between neuropsychologists and non-neuropsychologists in our sample; there were some differences in practice setting, and more neuropsychologists were non-Hispanic White, and more psychologists/mental health providers were Hispanic/Latino (see **Table 7**; **Supplementary Table S5**).

**Table 7:** Comparison of psychologists/mental health providers and neuropsychologists: demographics.

	Provider type									
	Psychologists / MH providers			Neuropsychologists						
Characteristic	*n*	*M*	*SD*	*n*	*M*	*SD*	*F* (df)	*η_p_^2^*	*p*	*p_Holm_*
Age	97	41.8	11.7	161	44.7	11.1	3.92 (1,256)	0.02	.049	.098
Years in practice	109	10.8	10.2	177	12.7	10.8	2.08 (1,284)	0.01	.150	.150
% FND patients	101	9.1	14.4	174	15.7	17.4	**10.28 (1,273)**	**0.04**	**.002**	**.006**

	*n*	%		*n*	%		*χ^2^*(df)	*z*	*p*	*p_Holm_*
Gender / sex (*n* = 276)							2.15 (2)		.342	–
Female	77	75.5		118	67.8		*-*	–	–	–
Male	24	23.5		55	31.6		*-*	–	–	–
Ethnicity / race (*n* = 265)							19.12 (4)		<.001	-
Ethnicity / race (*n* = 265)							**19.12 (4)**		**<.001**	**-**
Non-Hispanic White	66	68		149	88.7		*-*	4.1	**<.001**	**.005**
Asian / Asian American / Pacific Islander	11	11.3		9	5.4		*-*	1.8	.072	.216
Hispanic / Latino	10	10.3		3	1.8		*-*	3.1	**<.001**	**.005**
Black / African American	4	4.1		2	1.2		*-*	1.5	.134	.268
Biracial / Multiracial	6	6.2		5	3.0		*-*	1.3	.194	.268
Practice setting: Environment^a^ (*N* = 286)										
Private practice	63	57.8		51	28.8		**23.64 (1)**	**-**	**<.001**	**.006**
Academic hospital	16	14.7		65	36.7		**16.15 (1)**	**-**	**<.001**	**.006**
Government facility	18	16.5		24	13.6		0.47 (1)	–	.493	.954
Community hospital	5	4.6		29	16.4		**8.96 (1)**	**-**	**.003**	**.012**
Community practice	6	5.5		2	1.1		4.75 (1)	–	.029	.087
Other	10	9.2		21	11.9		0.51 (1)	–	.477	.954

Percentages were derived separately for each provider type. For each major category, family-wise corrections for multiple tests used the Holm-Bonferroni procedure. Statistics and *p*-values in bold indicate significant differences between groups after Holm adjustment. Percentages across a major category may not sum to 100 due to missing data or participants preferring not to answer. *η_p_^2^*, partial eta-squared; *p_Holm_*, Holm-adjusted *p*-values; for categorical variables, *z*-values represent adjusted standardized residuals for *post hoc* pairwise comparisons of proportions.

^a^ Because participants could choose more than one practice environment, the overall chi-square was not interpreted for this major category, which was instead tested independently.

Neuropsychologists reported a greater percentage of FND patients in their practice (**Table 7**) and demonstrated a greater understanding of FND based on coding of their open-ended definitions than did psychologists/mental health providers. Notably, nearly all of the providers who indicated “I don’t know” regarding the FND definition were in the psychologist/mental health provider category (See **Table 8**).

**Table 8:** Comparison of psychologists/mental health providers and neuropsychologists: providers’ FND understanding based on their FND definition.

	Provider type									
	Psychologists / MH providers			Neuropsychologists						
FND understanding	*n*	*%*		*n*	*%*		*χ^2^*(df)	*z*	*p*	*p_Holm_*
Coding for all responses to request for definition (*N* = 285)							**75.15 (3)**		**<.001**	**-**
No definition (“don’t know") (code = 0)	18	16.5		2	1.1			**4.9**	**<.001**	**.004**
Minimal/weak understanding (code = 1)	41	37.6		19	10.8			**5.4**	**<.001**	**.004**
Moderate/”so-so” understanding (code = 2)	33	30.3		52	29.5			0.1	.920	.920
Strong understanding (code = 3)	17	15.6		103	58.5			**7.1**	**<.001**	**.004**

FND understanding	*n*	*M*	*SD*	*n*	*M*	*SD*	*F* (df)	*η_p_^2^*	*p*	*p_Holm_*
FND understanding (codes 0 to 3)	109	1.4	0.9	176	2.5	0.7	**65.78 (1,263)**	**0.26**	**<.001**	*-*

Percentages for each level of FND understanding were derived separately for each provider type. Family-wise corrections for multiple tests used the Holm-Bonferroni procedure. Statistics and *p*-values in bold indicate significant differences between groups after Holm adjustment. *η_p_^2^*, partial eta-squared; *p*_Holm_, Holm-adjusted *p*-values; for categorical variables, *z*-values represent adjusted standardized residuals for *post hoc* pairwise comparisons of proportions. .

Neuropsychologists perceived having had more adequate training in both neuropsychological and general psychological assessment for FND than psychologists/mental health providers; however, there were no group differences in perceived adequacy of psychotherapy training for FND, with both groups reporting relatively low mean ratings (see **Table 9**).

**Table 9:** Comparison of psychologists/mental health providers and neuropsychologists: perceived training preparedness and attitudes toward FND.

	Provider type									
	Psychologists / MH providers			Neuropsychologists						
Characteristic	*n*	*M*	*SD*	*n*	*M*	*SD*	*F* (df)	*η_p_^2^*	*p*	*p_Holm_*
Perceived training preparedness to provide services to FND^b^ (*n* = 279)										
Psychotherapy	105	2.7	1.3	174	2.7	1.3	0.04 (1,277)	0	.852	.852
Psychological assessment	105	2.6	1.4	174	4	1.1	**87.03 (1,277)**	**.24**	**<.001**	**.004**
Neuropsychological assessment	105	1.9	1.2	174	4.4	0.9	**404.93 (1,277)**	**.59**	**<.001**	**.004**
Medication management^a^	105	1.4	1	174	1.1	0.5	**8.02 (1,277)**	**.028**	**.005**	**.010**
Attitudes and beliefs about FND^b^ (*n*s = 255 to 279)										
Symptoms can improve	105	3.8	0.9	174	4	0.8	4.57 (1,277)	.016	.033	.132
Challenging	82	3.6	1.1	174	3.8	0.9	3.37 (1,254)	.013	.068	.144
Neuro/medical concern	83	3.3	1	174	3	1	3.93 (1,255)	.015	.048	.144
Confidence	105	2.7	1.2	174	3.1	1.1	**8.04 (1,277)**	**.028**	**.005**	**.035**
Enjoyable	82	3.0	0.8	173	2.7	0.9	6.04 (1,253)	.023	.015	.075
Negative expectations	87	2.4	1	175	2.8	1.2	**7.50 (1,260)**	**.028**	**.007**	**.042**
Needs medical provider	89	2.2	0.8	175	2.1	0.8	0.17 (1,262)	.001	.678	.678

For each major category of variables, family-wise corrections for multiple tests used the Holm-Bonferroni procedure. Statistics and *p*-values in bold indicate significant differences between groups after Holm adjustment. *η_p_^2^*, partial eta-squared; *p*_Holm_, Holm-adjusted *p*-values; for categorical variables, *z*-values represent adjusted standardized residuals for *post hoc* pairwise comparisons of proportions.

^a^ The group difference in perceived adequacy of medication management training became non-significant when the five psychiatrists in the mental health provider group were excluded. ^b^Scales ranged from 1 to 5 with higher values reflecting greater endorsement of the item.

Finally, with respect to attitudes toward treating FND (see **Table 9**), neuropsychologists reported more confidence in working with FND than psychologists/mental health providers, yet greater negative expectations. With statistical correction for multiple comparisons, there were not significant group differences in how challenging FND patients were perceived, in the belief that symptoms could improve, or in the concern that symptoms were actually medical or that a medical (instead of psychological) service provider should treat FND.

## 4 Discussion

Psychotherapeutic interventions are integral to FND treatment, yet psychologists and other mental healthcare providers may not have the training, nor awareness of the extent of unmet patient need, to be able and willing to support FND patients. This is especially relevant for patients with FDS, for whom psychotherapy is the primary treatment of choice. Guidelines for neurologists that include referring to clinicians for psychosocial or other intervention (14) may be challenging to implement without an adequate provider base, leaving well-intentioned neurologists at a loss.

The present survey study aimed to gain a better understanding of the perspectives and practices of psychologists, neuropsychologists, and other mental healthcare providers in the U.S. with respect to FND.

### 4.1 FND understanding and attitudes

Findings yielded encouraging results, demonstrating that knowledge about FND is evolving. Definitions included but extended beyond conversion disorder and medically unexplained symptoms. Providers overall reported interest in gaining more knowledge about FND. They also reported a strong belief that FND symptoms can improve, as shown in both their quantitative and open-ended responses. Prior literature has often described providers’ stigmatizing beliefs and attitudes as a primary obstacle to successful FND healthcare experiences. However, there is also evidence, including the relatively low mean ratings and infrequent open-ended reports of such attitudes in our sample, that ideas about FND are changing (52). Although some negative attitudes were expressed in participants’ open-ended comments, they focused largely on systemic issues rather than patient-specific ones. Open-ended comments also conveyed both empathy for patients’ experience and a level of optimism regarding the potential for FND symptoms to improve with psychological treatment.

These results are novel and do not merely echo prior research that reported negative provider perceptions of FND. Rather, they parallel themes of “frustration and hope” found in previous qualitative work (44). In addition to a possible shift in attitude across the board as providers gain more awareness of FND (with greater guidelines for neurologists in addressing FND as noted above), our results may suggest that mental health care provider attitudes may be less negative than medical providers (39). This is particularly encouraging, because when providers treating FND hold less stigmatizing attitudes and feel more engaged in their clinical services to FND patients, improved treatment outcomes are likely. Positive attitudes also are important, given that most psychologists have a choice as to whether to take on FDS patients or to serve as part of an interdisciplinary team to treat FMD patients (i.e., whereas those working in a neurology milieu may not have such a choice).

Our data revealed an absence of contact or interaction with FND patients among psychologists/mental health providers as potentially problematic, as it may self-perpetuate the cycle of less knowledge, greater reluctance, and fewer available providers. Findings revealed that a larger percentage of FND patients in one’s practice and greater perceived preparedness to provide psychotherapy to FND – and not simply extent of knowledge about FND – were associated with fewer negative expectations and more enjoyment working with FND patients. Knowledge may be a critical starting point, but does not necessarily translate into positive attitudes and a willingness to take on patients with FND in one’s practice. Instead, providers who begin with a greater inclination to work with this population may be able to better anticipate the value and benefit of working with them; reciprocally, actively working with FND patients may promote more positive views and attitudes. Providers can further enhance the therapeutic alliance by understanding illness perception in FND patients, acknowledging their perceived lower control over their disorder, and by providing psychoeducation regarding the biopsychosocial complexities of FND (57).

### 4.2 Training barriers

Unexpectedly, lack of access to training per se was not reported as a barrier to gaining more knowledge about FND. Rather, the high ratio of cost (in terms of time and investment) to benefit was a notable barrier for providers who were only treating a small percentage of FND patients, or none at all. Even providers describing an interest in making such an investment noted that there was a lack of access to FND patients to further develop their treatment skills. In other words, whereas in certain medical settings (e.g., epilepsy monitoring units, movement disorders clinics) FND patients are present irrespective of the provider’s expertise and interest, paradoxically, psychologists may not regularly – or ever – encounter FND patients, and therefore it may require greater effort to learn how to provide treatment for this population most effectively.

### 4.3 The role of mental health providers: provision of psychotherapy

Close to half of our sample reported conducting psychotherapy as the service provided to FND patients, either alone or with assessment. Consistent with prior literature suggesting CBT is the primary type of treatment for FND (FDS and, where applicable, FMD), the majority of participants in our sample offering psychotherapy reported using CBT with FND patients, over half with an additional treatment modality (e.g., mindfulness-based, psychodynamic). This finding is encouraging, given that current recommendations for enhanced CBT, in the form of neurobehavioral therapy (NBT), combines components from multiple theoretical orientations. Based on their open-ended responses, providers are clearly drawing on a range of approaches – from psychoeducation to music therapy to family therapy – to address and/or treat FND. Continuing to not only develop new treatment protocols (e.g., such as NBT), but also to disseminate established protocols more widely and systematically; to provide guidance on how best to tailor them to unique patient needs; and to convey how clinicians can leverage skills they already have to treat FND are crucial next steps for the field.

### 4.4 The role of neuropsychologists: a bridge between neurology and psychology

The neuropsychologists in our sample reported more knowledge of FND, greater perceived training preparedness, and greater confidence in working with FND than the non-neuropsychologists/mental health providers. At the same time, neuropsychologists reported more negative expectations than non-neuropsychologists about working with FND. As we initially speculated, it is possible that closer contact with neurologists and other medical providers is associated with greater confidence and perhaps also greater stigma in attitudes toward FND. The nature of assessment itself (e.g., specific measures administered, short-term interaction with the client) is typically more structured and standard than ongoing therapy, which may result in more preparedness and confidence even when working with a complex condition such as FND.

Neuropsychologists’ knowledge of FND may be particularly important in the early stages of the patient’s understanding of their diagnosis and of treatment options, at the time when they are initially (or subsequently) assessed. As participants noted in their open-ended responses, presenting recommendations during an assessment feedback session also is an opportunity to educate other family members/caregivers (for pediatric or adult patients) about FND. Furthermore, neuropsychologists (or anyone conducting assessments) who use a testing battery and intake questions specifically for FND can provide essential information that will guide the treatment provider. Using performance validity tests during neuropsychological evaluation can enhance standard battery results and guide treatment by distinguishing between subjective and objective cognitive complaints (58). This further equips providers to select interventions (i.e., therapy modalities) that target specific psychological factors that may contribute to the cognitive performance results, allowing for opportunities for improvement in both domains. For example, patients found to have psychological trauma and PTSD symptomatology may be best treated with trauma-informed treatment. Collectively, although neuropsychologists may not be directly treating FND patients with these modalities, they can assist in guiding multidisciplinary treatment and goal setting due to having a distinct understanding of the patient’s cognitive profile, as reviewed in (59)]. As functional cognitive disorder (FCD) becomes better known (60, 61), neuropsychologists may play an increasingly pivotal role in differential diagnosis.

Approximately 28% of the neuropsychologists in our sample were providing psychotherapy to FND patients. Multiple participants suggested that neuropsychologists could be more involved in therapy or interdisciplinary treatment for FND. We echo the role of neuropsychologists as an important bridge between neurologists, who are the providers encountering and diagnosing FND patients, and community mental health providers, who conduct therapy but would not routinely encounter FND. There is also an opportunity for neuropsychologists to enter the referral pipeline by providing more FND treatment, and even to spearheading interdisciplinary treatment clinics as noted below.

### 4.5 Limitations

There are several notable limitations of this study, including sampling bias and potential lack of generalizability. We focused on knowledge, training, and attitudes of providers within the U.S., which may not reflect those of providers in other countries given likely healthcare system infrastructure and cultural differences. However, we cast a broad net by recruiting mental health providers across the U.S. and in a variety of practice environments to maximize representativeness with respect to practice characteristics. We also emphasized in our recruitment materials that providers did not need to be familiar with FND to take the survey. Consequently, many of our providers did not regularly encounter or work with patients with FND. We believe this reflects the general population of psychologists/mental health professionals, consistent with our initial speculation, although this cannot be confirmed based on the present design. At the same time, it is likely that we attracted clinicians for whom FND is more salient. Perhaps reflecting the salience of FND to certain sub-disciplines, we had a high proportion of neuropsychologists in our sample, but fewer than anticipated mental health providers/therapists, especially from disciplines such as counseling and clinical social work. Thus, in our subgroup comparisons we combined across the non-neuropsychology groups, as these specialties are similar overall in their focus on psychotherapy during training, rather than on assessment of neurocognitive functioning.

Further, 29% of participants overall (and 41% of neuropsychologists in our sample) reported having had an internship or fellowship with an epilepsy or movement disorders rotation, which may be higher than the general population of mental health/neuropsychologist practitioners. We did not assess whether participants had different training and practice experiences with subtypes of FND, such as FMD versus FDS. Given that treatment differs in important ways for these subtypes, it would be important in future studies to be more differentiated regarding how psychotherapists are delivering therapy in an individual context (more common for FDS) or integrating with multidisciplinary teams (e.g., therapies for movement, sensory, speech, and swallowing difficulties that are more likely to characterize FMD).

Another limitation of our study is the use of self-report, which is subject to social desirability bias even with anonymous questionnaires. This may be especially the case when assessing undesirable attitudes (e.g., negative perceptions of FND). Additionally, survey items were adapted from prior literature investigating FND-related attitudes, beliefs, and training among providers from other health care specialties (e.g., neurology, psychiatry, nursing, physical therapy), and those studies primarily used single items (40, 43, 48–55). While the current authors were guided by domain-related norms in terms of the approach and specific items they used (e.g., one study we used as a model consulted with the FDS task force of the International League Against Epilepsy [ILAE] when designing their questionnaire (53) survey items were not subjected to formal psychometric validation, potentially limiting reliability and replicability of the results.

There were also limitations with respect to specific items. For example, with respect to training attitudes, we asked, “How adequate was your training in preparing you to treat FND?” Ratings, therefore, may have reflected either FND-specific training (e.g., psychotherapy for FND), and/or perceptions of training in general (e.g., in psychotherapy), which respectively allowed participants to feel equipped (or not) to treat FND. Importantly, we do not have information on attitudes (e.g., confidence, enjoyment, negative expectations) in working with patients without FND. Consequently, comparisons of neuropsychologists and psychologists/mental health providers (e.g., greater confidence among neuropsychologists) may reflect general differences between these two groups, rather than FND-specific ones. Finally, the coding system used to categorize definitions of FND was developed for this study. Therefore, although it is face-valid, its reliability and generalizability are untested.

### 4.6 Implications and recommendations

Our study is one of the few to focus on mental health care providers’ knowledge of and attitudes toward FND. We recruited clinicians nationally within the US and across practice settings, which allowed us to assess perceptions among clinicians on the potentially receiving end of FND referrals, versus solely those embedded in FND and/or medically/neurologically-focused clinics.

Our findings underscore the importance of collaboration among psychologists/mental health providers, neuropsychologists, neurologists, and other specialists to exchange knowledge and coordinate medical and mental health treatment (62, 63). There are already a small but growing number of FND clinics embedded in inpatient and outpatient settings, and these serve as important treatment models. Gilmour et al. (9) suggest creating an FND clinic that shares similarities with other complex care centers, and their paper provides an example of a clinic structure for patients with FMD. Barbey et al. (64) provides an overview of their first Swiss FND clinic (both inpatient and outpatient settings). They suggest an interdisciplinary approach to treatment with individualized treatment for each patient. Finkelstein et al. (65) provide guidelines for setting up an FND interdisciplinary clinic that offers multiple therapies to FND patients, which aims to reduce costs, save time, and increase consistency for patients. These clinics are not a “one size fits all” approach but rather allow for adaption to changing FND symptoms over time (65).

Nevertheless, as establishing infrastructure for FND treatment is still in its infancy in many geographic areas, community mental health providers are needed for continued mental health treatment (especially for those with FDS, where stand-alone psychotherapy is recommended). To facilitate transition of care, based on our findings we suggest collaboration between the diagnosing neurologists or clinic and mental health providers, specifically targeting those providers (50-75% in our study) who are relatively interested in further FND training. This could involve a specified team leader (e.g., neurologist, neuropsychologist, psychiatrist, psychologist) who will facilitate these referrals to community mental health providers willing to accept FND patients into their practices and clinics. Ongoing collaboration with neurologists may assuage initial or downstream concerns held by mental health providers about the symptoms being medical, and would offer someone they can contact during a potential medical emergency.

Additionally, it would be beneficial for mental health providers to have access to an established network of providers who offer supervision and/or consultation to mental health providers treating FND. Clinicians interested in working with FND patients and/or providing consultation about FND can designate in marketing materials that they have expertise in this area (41). Another option for providers is listing their practice as part of available resources (e.g., the “Expert Information and Resources” page of www.nonepilepticseizures.com [managed by co-author LM]).

As our participants’ FND definitions highlight, effective referrals to mental health providers would likely benefit from bridging the gaps in terminology and conceptualization among conversion disorders, dissociative disorders, and FND (66). One consideration is that there may be a lack of knowledge regarding the terminology of FND added to the *DSM-5* (i.e., Functional Neurological Symptom Disorder). Providers may feel better equipped to treat patients if the referral conveys that the patient has “somatic symptoms.” Providers also may be more familiar with the terminology of “conversion disorder,” as was the case for a number of participants in our sample. However, this term has been associated with stigma and/or psychoanalytic modalities, and therefore newly-trained clinicians – especially in cognitive-behavioral approaches – may need yet other language to understand the context of the patient’s symptom presentation.

A key step is helping clinicians recognize that they already possess a strong skillset that they can use to treat FND, because the integration of even a small number of FND patients into one’s clinical practice may result in a meaningful difference in treatment availability. Treatments that focus on enhancing emotional awareness and distress tolerance (e.g., dialectical behavior therapy) or re-interpreting physical sensations (e.g., CBT for anxiety) are already adapted for FND. This is consistent with the integration of different theoretical orientations and approaches in NBT, an evidence-based treatment for FDS. There is mounting evidence for the efficacy of prolonged exposure therapy for FDS; because this treatment is widely known and practiced (given high prevalence of trauma and PTSD), it may be a readily-available option to address FDS symptoms (29). Additionally, group therapy with FND (embedded with other neurorehabilitation therapies) may be a promising intervention when it combines psychoeducation, aspects of CBT and emotion regulation, and a biopsychosocial framework to improve coping (67).

Finally, providing training experiences to clinicians through practicum placements at sites that provide psychotherapy to FND patients, adding continuing education on FND to national and international conferences, and providing internship and fellowship training to mental health providers in centers that offer a multidisciplinary approach to treatment can prepare mental health providers, including neuropsychologists, to feel confident in providing psychological treatment to FND patients.

In sum, the psychological community is in a strong position to establish larger networks of mental health providers who can treat FND by providing education and training experiences at the graduate and post-graduate levels (e.g., coursework, practica, internships, fellowships). Psychologists/neuropsychologists in medical settings who provide assessment and/or treatment for FND can foster collaborations with other disciplines such as neurology and cultivate a community network of mental health providers able to recognize and meet the FND treatment need.

## Data Availability

The original contributions presented in the study are included in the article/**Supplementary Material**. Further inquiries can be directed to the corresponding author.
