# Supplementary material for: Attitudes toward treating functional neurological disorders: psychological service provider perspectives
Source: Front Psychiatry. 2026 Oct 23;17:1892903. doi: 10.3389/fpsyt.2026.1892903 (PMC13740990; doi:10.3389/fpsyt.2026.1892903)
Supplement: Supplementary file 1 [file SupplementaryFile1.docx]

**Supplementary Material**

Included in this section:

- Supplementary Material A: *Definitions of FND: Category Descriptions and Examples*
- Supplementary Material B: *Open-ended Response Themes and Examples*
- Supplementary Tables S1 through S5:
  - Table S1. *Provider* *Clinical Practice Characteristics*
  - Table S2. *Providers Offering Psychotherapy, Psychological Assessment, or Neuropsychological Assessment for FND Patients*
  - Table S3. *Psychotherapy Modalities Provided*
  - Table S4. *Functional Neurological Disorder (FND) Training Experiences*
  - Table S5. *Comparison of Psychologists/Mental Health Providers and Neuropsychologists: Services Provided and Practice Characteristics*

**Supplementary Material A:**

**Participants’ Definitions of FND: Category Descriptions and Examples**

**(Corresponds to *Section 2.2.2, FND Definition Knowledge* and *Section 3.1.1, Knowledge of FND: Definitions*)**

As described in the Measures, participants’ responses to the question: “*To your knowledge, what is a ‘functional neurological disorder’ (FND)? Also, to your knowledge, what are some examples? If you do not know, please say don't know.*” were coded as 0 (don’t know), 1 (minimal/weak understanding), 2 (moderate understanding), or 3 (strong understanding). Only responses where the participant did not venture a guess were coded as 0. Any meaningful response or educated guess (even an inaccurate guess) was assigned a value from 1 to 3.

Definitions Reflecting Strong Understanding of FND (Coded as 3)

Definitions in this category typically captured a sophisticated, multifaceted understanding of FND, such as a bridge from its original conceptualization as a conversion disorder to a current conceptualization as a disruption in integration of motor, sensory, cognitive, and/or emotion-related processes. Although the concept of medically unexplained symptoms as relevant to FND was not in itself considered a strong definition, it often was included in a way that helped convey the participant’s understanding of the phenomenon. Examples are as follows:

“*A functional neurological disorder is characterized by sensory, motor, or cognitive symptoms that are significantly impacting day-to-day life, cause significant concern, and are not thought to be associated with an underlying medical condition. Typically, many medical tests have been performed which offer no alternative etiology for the symptoms. Psychogenic nonepileptic seizures are an example of this.*”

“*Patient is having neurological symptoms that do not have any evident medical/neurological basis nor consistent with known pathophysiology. Symptoms are not voluntarily controlled by patient. Typically requires comprehensive examination and diagnostics to rule out other possible disorders. Previously known as conversion disorder.*”

“*Newer broader term for what we used to called conversion d/o. often when neurological complaints can't be explained by known disease/medical process. Symptoms experienced are real, cause functional impairments and some believe there is a mis-firing from brain, etc, etc.*”

Definitions Reflecting Moderate Understanding of FND (Coded as 2)

Definitions in this category reflect a moderate understanding of FND. They are not as sophisticated as definitions coded as a 3, nor as inaccurate or vague as definitions coded as a 1, but rather fall in the range in-between these two anchors. Examples are as follows:

*“A condition with presentation for which structural/labs/medical explanations are unavailable and/or in which symptom presentation is beyond what can be explained medically”*

*“A disorder with neurological symptoms, that is medically unexplained, and partly or entirely psychogenic”*

“*NES, FMD, underlying trauma manifestations of somatic, mood, and memory difficulties”*

Definitions Reflecting Minimal or Weak Understanding of FND (Coded as 1)

Definitions coded in this category may either be inaccurate or may reflect a limited understanding of FND and its current conceptualization in the field. Examples are as follows:

“*I have never heard the specific term FND, but I assume it is any neurological disorder that allows the person to still function - ADHD, Autism, learning disability, head injury, etc.*”

“*not sure - used to be conversion DO.*”

*“a disorder in the brain that impairs functioning”*

*“FNDs are neurological conditions such as migraines, epilepsy, tics, etc. conditions that can be debilitating based on severity but also functional.”*

Definitions tended to repeat certain themes or terms, which we categorized and presented in Table 2 of the main document. Some of these categories were selected *a priori* (i.e., expectation of mention of conversion disorder, malingering, the term “pseudo,” and as a disorder of exclusion) and others were data-driven (i.e., mention of cognitive symptoms or functional cognitive disorder, FND as a disorder of inclusion or with identification via positive signs, and mention of other non-FND-specific psychological or medical terms/disorders). Participants likened FND to a number of other psychological and medical conditions (either correctly or incorrectly) or speculated as to associations among conditions and FND. These included, for example, chronic pain, fibromyalgia, traumatic brain injury, migraines, vision changes, or blindness (functional or medical), and autism spectrum disorders.

**Supplementary Material B:**

**Participants’ Perspectives about FND: Open-ended Response Themes and Examples**

**(Corresponds to *Section 2.2.4, Attitudes toward FND* and *Section 3.2.3, Provider Attitudes and Perspectives in Open-Ended Responses*)**

As described in the Measures, participants were given the opportunity to respond to the following prompt in an open-ended fashion: “*Please indicate anything else you would like to mention regarding working with patients with FND*.” Themes that stood out from these responses (i.e., based on multiple participants reporting a similar sentiment) and examples of comments reflecting each are listed below.

Many of the responses reflected **patient-level challenges** consistent with previous literature: Lack of acceptance of the diagnosis (“*It becomes challenging when they are resistant to accepting the diagnosis*”); comorbidities including personality disorders (“*often have co-occurring personality disorder traits and emotion regulation problems*”); and assumptions or witnessing others’ assumptions of malingering (“*Perceptions from clinicians of ‘malingering’ are also barriers to assisting the patient in getting the help they need.*”).

However, there appeared to be an overwhelmingly systemic/broader landscape emphasis from our sample. We focus on the latter perspective below, and also incorporate the perspective of those who do not work regularly (if at all) with FND.

First, consistent with our initial expectations and with the quantitative ratings suggesting relatively low perceived preparedness to treat FND with psychotherapy, participants’ open-ended responses revealed that they perceived the treatment landscape (i.e., irrespective of their own knowledge) as reflecting a **lack of providers with knowledge about FND and treatment options.** Comments included, “*We need MORE psychologists who feel comfortable treating FND*” (capitalization included in original response); “*Few places to refer them*”; and “…*it is a poorly served population which many practitioners avoid*.” One provider summarized: “*it is hard to be confident in the unknown*.”

More specifically, several respondents stated or implied that there was a **lack of established and/or readily available treatment protocols for FND.** For example, one commented: “*There are validated CBT treatments for FND but it is very hard to find psychotherapists who are familiar with these treatments. A single manualized treatment that is easily accessible online would be a great benefit. I have had trouble finding this other than books without clear treatment steps.*” Another commented: “*I wish that I had more support and training, as of now I do my own research to ensure my knowledge and use of empirically based treatment.*” A common sentiment was, “*Would love to see the development of more treatment protocols*.”

Other participants noted a **lack of available guidance in addressing patient heterogeneity or individual presentations**, and a lack of treatment success stories to draw upon. One participant noted, for example: “*I wish I could observe more about treatment for FND because I wonder if my perception is skewed with my one experience. In theory, the treatment makes sense to me and I am comfortable with it but it is hard to find examples of successful treatment stories and what that looks like for people. My client also always seems like they have a lot of barriers to getting better and I’m never sure if how much of that is because of her or FND.*”

Related, several participants pointed to a **low base rate of FND**, or infrequent contact with FND patients, which they reported made gaining knowledge and understanding difficult. At the same time, participants noted an uptick in their practice and/or other settings where they encountered FND patients. Responses (to this question and throughout the survey) pointed to **incidental rather than systematic FND knowledge.** One participant noted: “*I've only had one client with an FND and didn't even realize they had it until I read y'all's definition. I don't think they know either because their doctor just told them ‘We don't know why you're having seizures-must be a psychological thing.’*” Another noted, “*I don't work with anyone with an FND but I do have clients with physical manifestations of anxiety, such as a tremor of the right hand with anxiety...which is close to an FND I suppose.*” And yet another commented, “*To my knowledge, I am not aware of the proportion of my patients with FND.*” Thus, providers may encounter and even actively work with individual FND patients, yet may not conceptualize and/or label the symptom presentation as such.

Other providers similarly noted single FND cases they encountered throughout their career, especially while evaluating or treating other conditions – often in the context of residential facilities or clinics dedicated to those other conditions – such as epilepsy clinics, autism clinics, residential facilities for Alzheimer’s and other dementias, and pain clinics. Responses suggested that participants may not have reflected upon these experiences until taking the present survey.

Participants’ responses reiterated treatments that they adopted when working with FND, such as ACT, CBT, EMDR, family therapy (especially when working with youth with FND), and “somatic therapies.” Several mentioned psychological trauma as an etiology and/or comorbidity (“Just like any somatic or conversion disorder, treat the underlying cause -- depression, anxiety or trauma -- and the FND improves.”)

As a consequence of the lack of FND patients encountered routinely (versus, for example, patients with depression or traditional presentations of anxiety), **psychologists/mental health providers can choose not to engage** with the FND landscape. Examples include: “*Not in my area of practice*,” and “*Seeing as I don't work with patients with FND these questions are not relevant to me.*” Responses hinted at the fact that there may be a parallel process of isolation or stigma among those who do treat FND. Although framed positively, one provider captured, “*I'm an outlier as I run an FND treatment clinic.*”

Importantly, while the iatrogenic effects of misdiagnosis in FND are often discussed in the literature with respect to their direct impact on patient health (reviewed earlier), providers’ responses revealed concerns about **iatrogenic effects of the assessment and diagnostic process itself with respect to ease or effectiveness of psychological treatment.** As one participant captured: “*The most difficult part of working with patients with FND is the attitude of other professionals working with them and the iatrogenic impact of having been seen by multiple professionals who are lacking in understanding of and skills for diagnosing and treating these patients.*” Another noted, “*Interesting population that often have symptoms persist due to mismanagement by medical/mental health providers.*”

Providers also were concerned that further tests – which in some cases may be warranted – could exacerbate the lack of acceptance of psychological treatment. For example, in addition to noting the possibility of anxiety-related tremor, the provider quoted above explained: “*There's a lot of connected health anxiety which can be challenging to navigate since I can't give medical advice but also don't want to collude with the anxious thinking by having them get checked out by a medical provider constantly (they have already been cleared by their PCP).*”

This highlighted a genuine conundrum, especially if a differential diagnosis of FND has not yet been established, or when a mental health provider has concerns about the need to rule out medical causes for any symptom, regarding continuing that process yet without creating patient confusion or negating the need for psychological treatment.

Perhaps highlighting a shift in attitudes over time, participant responses underscored the **need for multi/interdisciplinary treatment and treatment teams**. Comments included: “*They definitely require a multidisciplinary team including at minimum psychiatry, neurology, and psychology.*” “*Interdisciplinary approach is needed that includes psychosocial supports and family involvement, as well as rehabilitation services if needed.*” “*The value of establishing a supportive, honest, and collaborative interdisciplinary therapeutic alliance.*” and “*With consistency in treatment and a multidisciplinary team we have the best outcomes.*”

Finally, given the high proportion of neuropsychologists in our sample, there were several responses mentioning neuropsychologist-related themes, including the **role and** **importance of neuropsychology in FND treatment**. One comment reflected the difficulty in bridging neuropsychology and community mental health providers: “*Much of my hesitation in completing neuropsychological assessments with individuals who have FND is lack of appropriate referral sources in the local community to help these individuals*.”

Some comments based on the perspective of neuropsychologists in our sample arguably were a “call to action” for neuropsychologists to work with FND: “*Neuropsychologists are specifically well-equipped to work with these patients*.” “*I believe more neuropsychologists need competencies in assessment and treatment of patients with FND.*” And “*FND is my therapy niche and what I enjoy the most (therapeutically). Neuropsychologists are the perfect people to treat this.*”

**Supplementary Tables**

| Table S1. *Provider* *Clinical Practice Characteristics* | | | |
| --- | --- | --- | --- |
| Characteristic | *n* | Subgroup %^a^ | Total %^b^ |
| Practice setting: Urbanicity | 253 |  |  |
| Urban only | 151 | 59.7 | 52.8 |
| Suburban only | 69 | 27.3 | 24.1 |
| Rural only | 24 | 9.5 | 8.4 |
| Multiple settings | 9 | 3.6 | 3.1 |
| Practice setting: Environment^c^ |  |  |  |
| Private practice | 114 | - | 39.9 |
| Academic hospital | 81 | - | 28.3 |
| Government facility | 42 | - | 14.7 |
| Community hospital | 34 | - | 11.9 |
| Community mental health practice | 8 | - | 2.8 |
| Other setting: Open-ended responses^d^ | 31 | - | 10.8 |
| Outpatient medical group/health center (including outpatient academic, community, or specialty settings) | 16 | - | 5.6 |
| Developmental disability center | 1 | - | 0.3 |
| Rehabilitation settings (including acute, neurological, and academic rehabilitation hospitals) | 6 | - | 2.1 |
| Psychiatric hospitals | 2 | - | 0.7 |
| Acute/long-term care facilities | 2 | - | 0.7 |
| Non-profit | 1 | - | 0.3 |
| Online practice | 1 | - | 0.3 |
| *Note*. Percentages may not add to 100 due to missing data/participants preferring not to answer or may exceed 100 where multiple options could be selected.  ^a^Subgroup % = percentage of participants who reported data for that category. ^b^Total % = percentage of full sample, whether or not they reported the category (*N* = 286). ^c^Participants were instructed to indicate their primary setting and to check multiple options if their time was split evenly among sites. ^d^Some participants listed more than one for practice environment “other setting” and some did not specify a location; four of the 31 also checked one or more of the provided options. | | | |

| Table S2. *Providers Offering Psychotherapy, Psychological Assessment, or Neuropsychological Assessment for FND Patients* | | | |
| --- | --- | --- | --- |
| Service provided | *n* | Subgrp % | Total % |
| Psychotherapy and/or assessment for FND: Unique individuals | 227 | - | 79.4 |
| Psychotherapy only | 52 | 22.9 | 18.2 |
| Psychological assessment only | 3 | 1.3 | 1.1 |
| Neuropsychological assessment only | 89 | 39.2 | 31.1 |
| Psychotherapy and psychological assessment | 15 | 6.6 | 5.2 |
| Psychotherapy and neuropsychological assessment | 15 | 6.6 | 5.2 |
| Psychological and neuropsychological assessment | 27 | 11.9 | 9.4 |
| Psychotherapy, psychological, and neuropsychological assessment | 26 | 11.5 | 9.1 |
| *Note*. Subgrp % = percentage of participants who reported providing psychotherapy or assessment for FND (*n* = 227). Total % = percentage of full sample, whether or not they reported the category (*N* = 286). | | | |

| Table S3. *Psychotherapy Modalities Provided* | | | |
| --- | --- | --- | --- |
| Psychotherapy modality | *n* | Subgrp % | Total % |
| Cognitive-behavioral therapy | 89 | 82.4 | 31.1 |
| Mindfulness-based therapy | 53 | 49.1 | 18.5 |
| Trauma-based therapy | 50 | 46.3 | 17.5 |
| Acceptance and commitment therapy | 40 | 37.0 | 14.0 |
| Dialectical behavior therapy | 22 | 20.4 | 7.7 |
| Psychodynamic therapy | 16 | 14.8 | 5.6 |
| Other psychotherapy modality^a^ | 26 | 24.1 | 9.1 |
| Analytic psychotherapy | 1 | 0.9 | 0.3 |
| Behavioral modification / habit reversal | 1 | 0.9 | 0.3 |
| Coping skills, distress tolerance | 2 | 1.9 | 0.7 |
| Eye Movement Desensitization and Reprocessing (EMDR) | 1 | 0.9 | 0.3 |
| Emotion-focused therapy / emotional awareness and expression therapy (EAET) | 3 | 2.8 | 1.0 |
| Family therapy / parent training | 2 | 1.9 | 0.7 |
| Gestalt psychotherapy | 1 | 0.9 | 0.3 |
| Group therapy | 2 | 1.9 | 0.7 |
| Hypnotherapy | 1 | 0.9 | 0.3 |
| Internal/Inner family systems therapy | 2 | 1.9 | 0.7 |
| Interpersonal therapy | 2 | 1.9 | 0.7 |
| Memory and Attention Adaptation Training (MAAT) | 1 | 0.9 | 0.3 |
| Music therapy | 1 | 0.9 | 0.3 |
| Narrative therapy | 1 | 0.9 | 0.3 |
| Person/client-centered therapy | 4 | 3.7 | 1.4 |
| Prolonged exposure | 1 | 0.9 | 0.3 |
| Psychoeducation, positive expectancies | 3 | 2.8 | 1.0 |
| Retraining and control therapy | 1 | 0.9 | 0.3 |
| Sex therapy | 1 | 0.9 | 0.3 |
| Short term dynamic therapy | 1 | 0.9 | 0.3 |
| Solutions focused therapy | 1 | 0.9 | 0.3 |
| Somatics | 1 | 0.9 | 0.3 |
| *Note*. Subgrp % = percentage of all participants who reported providing psychotherapy for FND patients either alone or in combination (*n* = 108). Total % = percentage of full sample, whether or not they reported providing therapy (*N* = 286). Across all items, percentage exceeds 100 because participants could select multiple options (e.g., participants selected trauma-based therapy, which was provided as an option, with some also writing in EMDR or prolonged exposure).  ^a^Yes-no question regarding other psychotherapy modality, followed by open-ended response. | | | |

| Table S4. *Functional Neurological Disorder (FND) Training Experiences* | | |
| --- | --- | --- |
| Training experiences | *n* | % |
| FND-specific training experiences | | |
| Read articles / books | 182 | 63.6 |
| Informal discussions with colleagues | 170 | 59.4 |
| Case conference / grand rounds | 143 | 50.0 |
| Professional conference presentations | 134 | 46.9 |
| Internship / fellowship with epilepsy or movement disorders rotation | 83 | 29.0 |
| Private supervision/consultation | 49 | 17.1 |
| Community-based trainings | 49 | 17.1 |
| Graduate program coursework | 41 | 14.3 |
| Practicum experience | 25 | 8.7 |
| Non-professional (personal) avenue / experience | 11 | 3.8 |
| Other training experiences^a^ | 37 | 12.9 |
| *Note*. Percentages are calculated from the full sample (*N* = 286). Across all items, percentage exceeds 100 because participants could select multiple options.  ^a^Most participants who selected “other training experiences” also selected other categories (*n* = 29 or 78%); descriptions of other training experiences largely overlapped with the categories provided but with more specificity (e.g., the specific conference attended). | | |

| Table S5. *Comparison of Psychologists/Mental Health Providers and Neuropsychologists: Services Provided and Practice Characteristics* | | | | | | | | | | | | | | | |
| --- | --- | --- | --- | --- | --- | --- | --- | --- | --- | --- | --- | --- | --- | --- | --- |
|  | Provider type | | | | | | | | | |  | |  |  |  |
|  | Psychologists / MH Providers | | | | |  | | Neuropsychologists | | | |  |  |  |  |
|  | *n* | | % | |  | |  | *n* | % |  | *Χ^2^* (df) | | *z* | *p* | *p_Holm_* |
| Provides psychotherapy and/or assessment for FND (*n* = 227) | | | | | | | | | | | **159.69 (6)** | |  | **<.001** | **-** |
| Psychotherapy only | 46 | 65.7 | |  | |  | | 6 | 3.8 |  | - | | **10.2** | **<.001** | **.007** |
| Psychological assessment only | 2 | 2.9 | |  | |  | | 1 | 0.6 |  | - | | 1.4 | .161 | .161 |
| Neuropsychological assessment only | 3 | 4.3 | |  | |  | | 86 | 54.8 |  | - | | **7.2** | **<.001** | **.007** |
| Psychotherapy and psychological assessment only | 14 | 20.0 | |  | |  | | 1 | 0.6 |  | - | | **5.4** | **<.001** | **.007** |
| Psychotherapy and neuropsychological assessment only | 1 | 1.4 | |  | |  | | 14 | 8.9 |  | - | | 2.1 | .036 | .072 |
| Psychological assessment and neuropsychological assessment only | 1 | 1.4 | |  | |  | | 26 | 16.6 |  | - | | **3.3** | **<.001** | **.007** |
| Psychotherapy, psychological, and neuropsychological assessment | 3 | 4.3 | |  | |  | | 23 | 14.6 |  | - | | 2.3 | .021 | .063 |
| Practice setting: Geographic region (*N* = 286) | | | | | | | | |  |  | **31.19 (5)** | |  | **<.001** | **-** |
| Southwest | 46 | 42.2 | |  | |  | | 29 | 16.4 |  | - | | **4.8** | **<.001** | **.006** |
| Midwest | 18 | 16.5 | |  | |  | | 53 | 29.9 |  | - | | **2.6** | **.009** | **.036** |
| West | 21 | 19.3 | |  | |  | | 28 | 15.8 |  | - | | 0.8 | .424 | 1.000 |
| Northeast | 15 | 13.8 | |  | |  | | 27 | 15.3 |  | - | | 0.3 | .764 | 1.000 |
| Southeast | 6 | 5.5 | |  | |  | | 32 | 18.1 |  | - | | **3.0** | **.003** | **.015** |
| Non-U.S. | 3 | 2.8 | |  | |  | | 8 | 4.5 |  | - | | 0.8 | .424 | 1.000 |
| Practice setting: Urbanicity^a^ (*N* = 286) | | | | | |  | |  |  |  | **-** | | **-** | **-** | **-** |
| Rural | 12 | 11 | |  | |  | | 26 | 14.7 |  | 0.79 (1) | | - | .373 | .746 |
| Suburban | 41 | 37.6 | |  | |  | | 68 | 38.4 |  | 0.02 (1) | | - | .892 | .892 |
| Urban | 79 | 72.5 | |  | |  | | 111 | 11.9 |  | 2.86 (1) | | - | .089 | .267 |
| *Note*. Percentages derived separately for each provider type. For each major category of variables, family-wise corrections for multiple tests used the Holm-Bonferroni test. Statistics and *p*-values in bold indicate significant differences between groups after Holm adjustment. Percentages across a major category may not sum to 100 due to missing data or participants preferring not to answer, or may exceed 100 where multiple options could be selected. *η_p_^2^* = partial eta-squared; *p_Holm_* = Holm-adjusted *p*-values; for categorical variables, *z*-values represent adjusted standardized residuals for post hoc pairwise comparisons of proportions.  ^a^Because participants could choose more than one urbanicity, the overall chi-square was not interpreted for this category, which was instead tested independently. | | | | | | | | | | | | | | | |
